# Supplementary material for: HIVprotI: an integrated web based platform for prediction and design of HIV proteins inhibitors
Source: J Cheminform. 2018 Mar 9;10:12. doi: 10.1186/s13321-018-0266-y (PMC5845081; doi:10.1186/s13321-018-0266-y)
Supplement: Supplementary file 2 — Additional file 2. Source code of HIVProtI web server. [file 13321_2018_266_MOESM2_ESM.zip › HIVprotI_Source-code/hivprotI/weka-3-6-6/doc/weka/attributeSelection/AttributeTransformer.html]

AttributeTransformer


---


|  |  |  |
| --- | --- | --- |
| **Overview** \| **Package** \| **Class** \| **Tree** \| **Deprecated** \| **Index** \| **Help** \| **Weka's home** | |  |
| **PREV CLASS**   **NEXT CLASS** | **FRAMES**    **NO FRAMES**     **All Classes** |  |
| SUMMARY: NESTED \| FIELD \| CONSTR \| METHOD | DETAIL: FIELD \| CONSTR \| METHOD |  |


---


## weka.attributeSelection Interface AttributeTransformer

**All Known Implementing Classes:**: LatentSemanticAnalysis, PrincipalComponents

---

``` public interface AttributeTransformer ```

Abstract attribute transformer. Transforms the dataset.

**Version:**
:   $Revision: 4613 $

**Author:**
:   Mark Hall (mhall@cs.waikato.ac.nz)

---

| **Method Summary** | |
| --- | --- |
| `Instance` | `convertInstance(Instance instance)`             Transforms an instance in the format of the original data to the transformed space |
| `Instances` | `transformedData(Instances data)`             Transform the supplied data set (assumed to be the same format as the training data) |
| `Instances` | `transformedHeader()`             Returns just the header for the transformed data (ie. |

**Method Detail**

### transformedHeader

```
Instances transformedHeader()
                            throws java.lang.Exception
```

:   Returns just the header for the transformed data (ie. an empty
    set of instances. This is so that AttributeSelection can
    determine the structure of the transformed data without actually
    having to get all the transformed data through getTransformedData().

    :   **Returns:**: the header of the transformed data. **Throws:**: `java.lang.Exception` - if the header of the transformed data can't be determined.

---


### transformedData

```
Instances transformedData(Instances data)
                          throws java.lang.Exception
```

:   Transform the supplied data set (assumed to be the same format
    as the training data)

    :   **Returns:**: A set of instances representing the transformed data **Throws:**: `java.lang.Exception` - if the attribute could not be evaluated

---


### convertInstance

```
Instance convertInstance(Instance instance)
                         throws java.lang.Exception
```

:   Transforms an instance in the format of the original data to the
    transformed space

    :   **Returns:**: a transformed instance **Throws:**: `java.lang.Exception` - if the instance could not be transformed


---


|  |  |  |
| --- | --- | --- |
| **Overview** \| **Package** \| **Class** \| **Tree** \| **Deprecated** \| **Index** \| **Help** \| **Weka's home** | |  |
| **PREV CLASS**   **NEXT CLASS** | **FRAMES**    **NO FRAMES**     **All Classes** |  |
| SUMMARY: NESTED \| FIELD \| CONSTR \| METHOD | DETAIL: FIELD \| CONSTR \| METHOD |  |


---
